# Inhibition of HIF-1α Attenuates Silica-Induced Pulmonary Fibrosis

**DOI:** 10.3390/ijerph19116775

**Published:** 2022-06-01

**Authors:** Xiao Xu, Yiping Li, Zhuoya Niu, Jiarui Xia, Kai Dai, Chen Wang, Wu Yao, Yonghua Guo, Xuedan Deng, Jing He, Meng Deng, Huifang Si, Changfu Hao

**Affiliations:** Department of Occupational Health and Environmental Health, College of Public Health, Zhengzhou University, Zhengzhou 450001, China; xxiao1995@hotmail.com (X.X.); 13608691126@163.com (Y.L.); niuzya@163.com (Z.N.); xiajiarui2022@163.com (J.X.); daikai0301@163.com (K.D.); wang2426500823@163.com (C.W.); yaowu@zzu.edu.cn (W.Y.); yonghuag163@163.com (Y.G.); dxd690805@163.com (X.D.); 15932291792@163.com (J.H.); dengmeng819025371@163.com (M.D.); huifang_si@163.com (H.S.)

**Keywords:** hypoxia-inducible factor-1α, silica, pulmonary fibrosis, myofibroblasts, transforming growth factor-β1

## Abstract

Background: Excessive accumulation of extracellular matrix is a key feature of pulmonary fibrosis (PF), and myofibroblasts are the main producers of extracellular matrix. Fibroblasts are the major source of myofibroblasts, but the mechanisms of transdifferentiation are unclear. Methods: In vitro, transforming growth factor-β1 was used to induce NIH-3T3 cell transdifferentiation. DMOG was used to increase hypoxia-inducible factor-1α subunit (HIF-1α) expression. KC7F2 and siRNA decreased HIF-1α expression. In vivo, silica particles were used to induce PF in C57BL/6N mice, and KC7F2 was used to reduce HIF-1α expression in C57BL/6N mice. Western blot was used to detect the expression of collagen type 1 alpha 1(COL1A1), α-smooth muscle actin (α-SMA), SMAD family member (SAMD) 3, Phospho-SMAD3 (PSMAD3), and HIF-1α. PCR was used to detect the expression of COL1A1, α-SMA, and HIF-1α. Immunohistochemistry was used to detect the expression of COL1A1 and HIF-1α. Results: In vitro, compared to the control group, COL1A1, α-SMA, PSMAD3, and HIF-1α expression were elevated in the DMOG group, and COL1A1, α-SMA, PSMAD3, and HIF-1α expression were decreased in the KC7F2 group and siRNA group. Compared to the DMOG group, COL1A1, α-SMA, and PSMAD3 expression were decreased in the DMOG + SIS3 group. In vivo, compared to the saline group, COL1A1, α-SMA, PSMAD3, and HIF-1α expression were increased in the pulmonary tissue of C57BL/6N mice in the silica group. Compared to the silica group, COL1A1, α-SMA, PSMAD3, and HIF-1α expression and the degree of PF were decreased in the silica + KC7F2 group. Conclusion: Inhibition of HIF-1α reduced α-SMA, decreased COL1A1 expression, and attenuated the degree of PF in C57BL/6N mice. Therefore, HIF-1α may be a new target for the treatment of silica-induced PF.

## 1. Introduction

Long-term inhalation of silica particles induces lung fibrosis and impairs lung gas exchange functions. As a result, many PF patients suffer from dyspnea. Silica particles inhaled into the lungs destroy the alveolar structure, causing impaired lung function. The silica particles are phagocytosed by alveolar macrophages, which leads to the death of alveolar macrophages. The dead alveolar macrophages secrete large amounts of inflammatory factors and cytokines, such as interleukin-6, tumor necrosis factor-α (TNF-α), transforming growth factor-β1 (TGF-β1), and connective tissue growth factor, which exacerbate the alveolar inflammatory damage [1] Myofibroblasts, as the main effector cells of PF, secrete large amounts of extracellular matrix (ECM), which can be formed by the transdifferentiation of fibroblasts. Myofibroblasts are the main effector cells of PF. The accumulation of myofibroblasts not only causes the secretion of ECM but also affects the development of PF. Myofibroblasts can be transformed by fibroblasts stimulated by TGF-β1 [2,3,4]. The massive deposition of ECM further impairs oxygen exchange in the alveoli and aggravates hypoxic symptoms. PF is an irreversible process, and the development of the disease can only be prevented or delayed. Some medications were considered to be effective in relieving PF but had side effects and were not very effective in treating PF. For example, nintedanib and pirfenidone are thought to reduce PF, but they have serious side effects. Jinshuibao capsules have been clinically discussed in the treatment of PF, and the results show that jinshuibao capsules have a good therapeutic effect. They can reduce the level of TNF-α and TGF-β1 to reduce inflammation, increase lung capacity and maximum spontaneous ventilation, and reduce airflow restriction [5]. In addition, some studies have explored the clinical efficacy of acetylcysteine combined with tetrandrine tablets in the treatment of PF. The results of Sun et al. [6] showed that the lung function of the two groups of patients improved after different treatments, the levels of serum interleukin-6 and TNF-α decreased, and the decrease degree in the observation group was significantly higher than that in the control group. This shows that tetrandrine combined with acetylcysteine can effectively improve the clinical treatment effect of silicosis and reduce the degree of inflammation in silicosis patients. Tang et al. [7] also found that Dahuang Zhechong pills (DHZCP) could improve lung function, quality of life, and exercise ability in PF patients. The lung damage caused by particulate matter is related to the type and particle size. For example, different particle sizes of silica can reach different areas and cause different types of damage to the body. In terms of oxidative damage, micron-sized silica particles mainly cause damage to the lungs, mostly deposited in the alveoli, while nanoscale-sized particles can reach many tissues in the body and cause damage such as damage to the interstitial lung, the circulatory system, and the reproductive system. Micron-sized silica particles deposited in the alveoli cause persistent damage to the lungs and eventually cause PF.

TGF-β1 can promote cell migration, proliferation, differentiation, morphological changes, secretion of ECM and tissue repair and has an essential role in the process of fibrosis [8,9]. TGF-β1 then activates TGF-β1 receptor type I kinase, leading to SMAD2 and SMAD3 phosphorylation. Subsequently, activated SMAD2 and SMAD3 form oligomeric complexes with SMAD4. These oligomeric complexes translocate to the nucleus, regulating the transcription of target genes [10]. Fibrotic diseases are accompanied by many growth factors, among which endogenous TGF-β1, which promotes tissue repair and wound healing, plays an important role. When cells are exposed to TGF-β1, it inhibits deptor and increases mTorC1 protein expression [11,12]. mTor has two protein complexes, mTorc1 and mTorc2. It has been shown that elevated levels of mTorc1 can promote the production of HIF-1α, leading to elevated expression of HIF-1α target genes, such as vascular endothelial growth factor (VEGF) and fibroblast growth factor [13,14,15]. The expression of HIF-1α is elevated in liver fibrosis and myocardial fibrosis caused by TGF-β1. PF induced by silica also causes an increase in TGF-β1. TGF-β1 upregulates HIF-1α expression. Thus, we attempted to establish a link between HIF-1α and silica-induced PF.

HIF-1α oxygen-dependent structural domain (HIF-1α ODD) contains two proline residues, P402 and P564, which can serve as substrates for the proline hydroxylase domain (PHD) [16,17]. In normal oxygen, the P402 and P564 residues on the HIF-1α ODD are hydroxylated by PHD [18]. The hydroxylated HIF-1α ODD increases the affinity to VonHippel–Lindau tumor suppressor protein (pVHL) and accelerates the ubiquitination of HIF-1α [19]. The ubiquitinated HIF-1α is recognized and rapidly degraded by the proteasome. The half-life of HIF-1α in normoxic environment is very short, about 5 min. In a hypoxic environment, PHD activity is inhibited, the affinity of HIF-1α ODD and pVHL are weakened, the ability of the proteasome to recognize and decompose HIF-1α is reduced, and the stability of HIF-1α is elevated. The nuclear localization sequence on the escaped HIF-1α binds to the nuclear pore protein and rapidly enters the nucleus and combines with the HIF-β isoform in the nucleus to form a dimer, which binds to the hypoxia response element site on DNA and causes the activation of the transcription of downstream genes. HIF-1α has been reported to play an important role in liver fibrosis, kidney fibrosis, and myocardial fibrosis, but its effect in PF is unclear.

The main effector cells of PF are myofibroblasts. Fibroblast transdifferentiation is an important source of myofibroblasts. Endogenous TGF-β1 promotes fibroblast transdifferentiation, but the mechanism is unclear. It has been shown that TGF-β1 upregulates HIF-1α expression via the mTOR pathway and that HIF-1α plays a role in fibrotic diseases. Therefore, this study was conducted to observe the effect of HIF-1α expression on fibroblast transdifferentiation by interfering with HIF-1α expression. These results further elucidate the role of HIF-1α in silica-induced PF.

## 2. Materials and Methods

### 2.1. Reagents

TGF-β1 (abs04222, absin, Shanghai, China) was purchased from Absin Co. (Shanghai, China). KC7F2 (IK0250, Solarbio, Beijing, China), (E)-SIS3 (SIS3) (IS1250, Solarbio), dimethyloxallyl glycine (DMOG) (ID0540, Solarbio), dimethy sulfoxide (DMSO) (D8371, Solarbio), dulbecco’s modified eagle’s medium (DMEM) (11995, Solarbio), Newborn calf serum (NBCS) (80230-6415, Solarbio) sodium pyruvate, bonessential amino acid solution were purchased from Solarbio (Beijing, China). The primary antibodies used in Western blot include SMAD3 (ab208182, Abcam, Cambridge, UK), PSMAD3 (9520S, 36169, Cell Signaling Technology, Danvers, MA, USA), α-SMA (ab179467, Abcam), COL1A1 (ab260043, Abcam), β-Actin (20536-1-AP, Proteintech, Wuhan, China), and HIF-1α (36169, Cell Signaling Technology) RNAiso Plus reagent was obtained from Takara Biomedical Technology (Kyoto, Japan). PrimeScript™ RT reagent Kit with gDNA Eraser (Perfect Real Time) and SYBR Green PCR kit was purchased from Takara Biomedical Technology (Kyoto, Japan). Silica particles were purchased from Sigma-Aldrich (St. Louis, MO, USA). Particle size distribution was made up of 90% < 5 μm diameter and 73% < 3 μm diameter, with a median diameter of 2.244 μm.

### 2.2. Cell Culture

NIH-3T3 fibroblasts cells were purchased from the cell bank of the Chinese Academy of Sciences (Shanghai, China). The cells were cultured in Dulbecco’s modified Eagle medium (DMEM) added with 10% NBCS, 1% nonessential amino acid, and 1% sodium pyruvate at 37 °C 5% CO_2_. When the cells reached 70–80% density, the cells were digested with 0.25% trypsin and inoculated in six-well plates at a density of 10,000 cells per cm^2^ and incubation was continued for 24 h at 37 °C 5% CO_2_. The cells were collected for subsequent analysis using different treatments and continued incubation for 24–48 h, depending on the group.

### 2.3. Modeling of Transdifferentiation

TGF-β1 (abs04222, absin) was purchased from Absin Co. (Shanghai, China). NIH-3T3 cells were inoculated in six-well plates at a density of 10,000 cells per cm2 and incubation was continued for 24 h at 37 °C 5% CO_2_ before establishing the transdifferentiation model. NIH-3T3 transdifferentiation models were established using 0, 2.5, 5, 7.5, and 10 ng/mL TGF-β1-stimulated cells for 24 h. NIH-3T3 transdifferentiation model was established by treatment with 7.5 ng/mL TGF-β1 for 0 h, 2 h, 4 h, 6 h, 8 h, 10 h, 12 h, 24 h, and 48 h.

### 2.4. Small-Molecule Intervention and Treatment of NIH-3T3 Cells

We purchased DMOG (ID0540, Solarbio), KC7F2 (IK0250, Solarbio), (E)-SIS3 (SIS3) (IS1250, Solarbio), DMSO (D8371, Solarbio) from Solarbio co (Shanghai, China) and dissolve DMOG, SIS3, and KC7F2 in DMSO according to the manufacturer’s instructions. NIH-3T3 cells were inoculated in six-well plates at a density of 10,000 cells per cm^2^ and incubation was continued for 24 h at 37 °C 5% CO_2_ before intervention. According to the different groupings, NIH-3T3 cell was treated with DMOG (20 μmol/L) or KC7F2 (20 μmol/L) or SIS3 (20 μmol/L) or DMOG (20 μmol/L) + SIS3 (20 μmol/L).

### 2.5. Transfection of Small-Interfering RNA

We purchased siRNA and control siRNA that match the selected region of HIF-1α from Genechem Co. (Shanghai, China). According to the manufacturer’s instructions, lipofectamine RNA-iMAX (Invitrogen, Carlsbad, CA, USA) reagent was used to transfect siRNA. NIH-3t3 cells were inoculated in six-well plates at a density of 10,000 cells per cm^2^ and incubation was continued for 24 h at 37 °C 5% CO_2_ before transfection. We mixed 125 μL of serum-free medium containing 20 μM HIF-1α-siRNA or NC-siRNA with 125 μL of serum-free medium containing 5 μL of RNA-IMAX reagent, placed the mixture at 37 °C for 10 min, and added serum-free DMEM medium for culturing cells. The cells were incubated in an incubator at 37 °C 5% CO_2_ for 6 h. The serum-containing medium was replaced, and the cell was treated differently according to the grouping. Cells were collected for the assay. Briefly, serum-free medium containing siRNA was mixed with serum-free medium containing RNA-IMAX reagent and added to serum-free medium, and cells were cultured for 6 h.

### 2.6. Animals

Male C57BL/6N mice (4 weeks of age, 16–18 g) were purchased from Beijing Vital River Laboratory Animal Technology Co., Ltd. (Beijing, China) Furthermore, at the College of Public Health, Zhengzhou University, they were raised in specific-pathogen-free conditions. All animal experiments comply with “Animal management regulations”.

### 2.7. Animal Model of Silicosis

Silica particles were purchased from Sigma-Aldrich (St. Louis, MO, USA). Particle size distribution was made up of 90% < 5 μm diameter and 73% < 3 μm diameter, with a median diameter of 2.244 μm. The silica particles were mixed with saline and vortexed to make silica-particle suspension. Forty C57BL/6N mice were randomly divided into the saline-treated group (saline group, n = 10), silica-treated group (silica group, n = 10), silica-treated with the KC7F2 intervention group (silica + KC7F2 group, n = 10), and silica-treated with intervention negative control group (silica + NC group, n = 10). Mice were placed in isoflurane-filled chambers for anesthesia, followed by tracheal dripping of 50 μL of silica-particle suspension (50 mg/mL) or saline and continued feeding. The saline group was treated with saline for tracheal drip. The remaining groups were treated with silica-particle suspension solution for tracheal drip. In the KC7F2 group, KC7F2 (0.75 mg/Kg) solution was injected intraperitoneally every 3 days. In the NC group, the same volume of saline was injected intraperitoneally every 3 days. After 28 days, the mice were completely anesthetized with 2% sodium pentobarbital solution and sacrificed and lung tissues were taken for testing.

### 2.8. RT-qPCR

The same batch of samples was used for RT-qPCR detection. According to the manufacturer’s protocol, the total RNA of cells and lung tissue was extracted using RNAiso Plus reagent (Takara, Kyoto, Japan). The quality and concentration of RNA were detected using NanoDrop 2000 spectrophotometer (Seamer Fischer Technology Co., Ltd., Wilmington, TX, USA). One ug RNA was extracted from each sample for detection. Reverse transcription RNA was performed using PrimeScript RT reagent Kit with gDNA Eraser (Perfect Real Time) kit (both purchased by Takara). The reverse transcription regimen lasted 15 min at 37 °C and 5 s at 85 °C. Then, the SYBR Green PCR kit (Takara, Kyoto, Japan) was used on a real-time PCR detection system (Quant-Studio 7 Flex, Thermo Scientific, Waltham, MA, USA). According to the actual manufacturer’s protocol, the thermal cycle condition is 95 °C for 30 s, followed by a 40 thermal cycle of 95 °C for 3 s and 60 °C for 30 s. The fold changes of gene expression were calculated using the 2^−ΔΔCT^ method by normalizing β-Actin. Each analysis was repeated three times using different donors. The primers used were provided by Sangon Biotech (Shanghai, China). The primer sequence is shown in Table 1.

### 2.9. Western Blot Analysis

Proteins were extracted from cells or lung tissue using RIPA lysis buffer (Beyotime, Shanghai, China) containing 1% PMSF with 1% phosphatase inhibitor (both obtained from Beyotime) according to the protocol provided by the reagent manufacturer. Protein concentrations were determined using the BCA protein assay Kit (Boster Biological Technology, Wuhan, China). Loading Buffer (LT101S, Epizyme, Shanghai, China) was added to the protein extraction solution, heated to 95 °C for 5 min, and stored at low temperatures. Proteins were transferred to PVDF membranes (Merck, MA, USA) for 2 h. Subsequently, the gels were closed for 2 h using 5% skim milk powder (BD, Franklin Lakes, NJ, USA) (dissolved in tris-buffered saline and supplemented with 0.1% tween-20 (TBST)). PVDF membranes were incubated overnight at 4° with primary antibodies against HIF-1α, SMAD3, PSMAD3, β-actin, COL1α1, and α-SMA genes. PVDF membrane was washed three times with 1× TBST and was incubated with secondary antibody at 37° for 2 h and then rewashed three times with 1× TBST. Proteins were detected using ECL detection reagent (Beyotime) and fluorescent signals were collected using Amersham Imager 600 system (GE, Fairfield, UK). To confirm that the sample sizes were equal, we used β-actin to detect the blots and analyzed them using ImageJ software.

### 2.10. Histology

The right lower lung of the mice was immersed in 4% paraformaldehyde for at least 24 h and embedded in paraffin. The lung tissue samples were cut into 5 μm thick sections for pathological analysis. Masson trichrome staining and HE staining evaluated the degree of PF.

### 2.11. Immunohistochemistr (IHC)

For IHC staining, refer to the papers [20]; 5 μm thick sections were incubated with rabbit anti-mouse primary antibodies against HIF-1α (1:200, 20960-1-AP, ProteinTech) and COL1A1 (1:1000, GB11022-3, Servicebio), followed by horseradish peroxidase (HRP)–conjugated goat anti-rabbit IgG secondary antibody (1:8000, SA00001-2, Proteintech).

### 2.12. Statistical Analysis

All the data were expressed as mean ± SD, and the statistical analysis was calculated with SPSS 21.0 software. The independent samples *t*-test was used to compare two groups, whereas one-way analysis of variance (ANOVA) was used to compare multiple groups and pairwise comparisons were compared by LSD-t test. A value of *p* < 0.05 was considered to be statistically significant.

## 3. Result

### 3.1. TGF-β1 Induces Cells Transdifferentiation in a Dose-Dependent Manner

**Figure 1:**
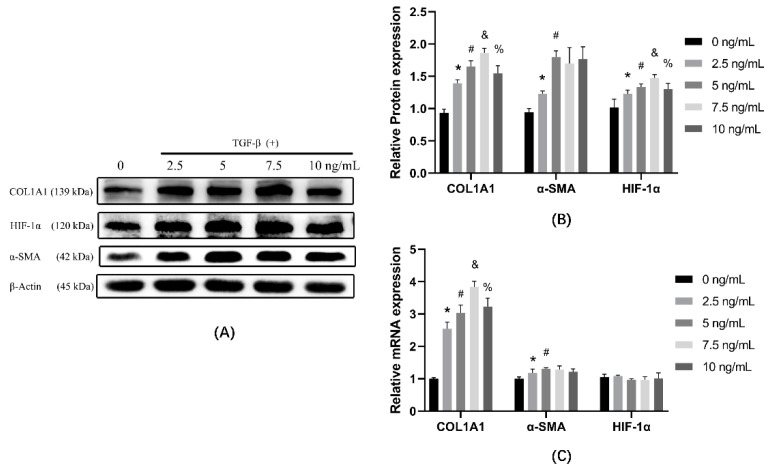
The effect of TGF-β1 on cell transdifferentiation was analyzed by comparing the expression of COL1A1, HIF-1α, and α-SMA using TGF-β1-treated cells at different concentrations. (**A**) Western blot analysis of col1a1, HIF-1α, and α-SMA in cells of different groups. β-actin was used as loading control. (**B**) Quantitative analysis of COL1A1, HIF-1α, and α-SMA is shown in Figure 1A. (**C**) Cells were treated with TGF-β1 at different concentrations, and mRNA was extracted from the treated groups. mRNA expression was determined by qPCR and normalized to β-actin. * *p* < 0.05 vs. 0 ng/mL group; ^#^ *p* < 0.05 vs. 2.5 ng/mL group; ^&^ *p* < 0.05 vs. 5 ng/mL group; ^%^ *p* < 0.05 vs. 7.5 ng/mL group. Results are expressed as means ± SD. ANOVA was used to compare multiple groups and pairwise comparisons were compared by LSD-t test. A value of *p* < 0.05 was considered to be statistically significant.

NIH-3T3 cells were treated with 0, 2.5, 5, 7.5, and 10 ng/mL of TGF-β1 for 24 h and subsequently collected and analyzed. As shown in the protein result (Figure 1A,B), HIF-1α showed a dose-dependent increase from 0 to 7.5 ng/mL and reached a maximum at 7.5 ng/mL concentration, followed by a slight decrease. The expression of α-SMA increased and was highest at 5 ng/mL, but the change was not statistically significant beyond 5 ng/mL. The expression of COL1A1 reached the maximum at 7.5 ng/mL, the same as the peak concentrations of HIF-1α. mRNA (Figure 1C) and protein results (Figure 1B) showed a similar trend, with α-SMA at 5 ng/mL and COL1A1 reaching the maximum at a 7.5 ng/mL peak. The mRNA of HIF-1α showed no alteration. TGF-β1 has been reported to cause an increase in HIF-1α translation via mTOR1 without affecting transcript levels; therefore, the absence of alterations in HIF-1α mRNA is consistent with this conclusion.

### 3.2. TGF-β1 Causes Cellular Transdifferentiation in a Time-Dependent Manner

**Figure 2:**
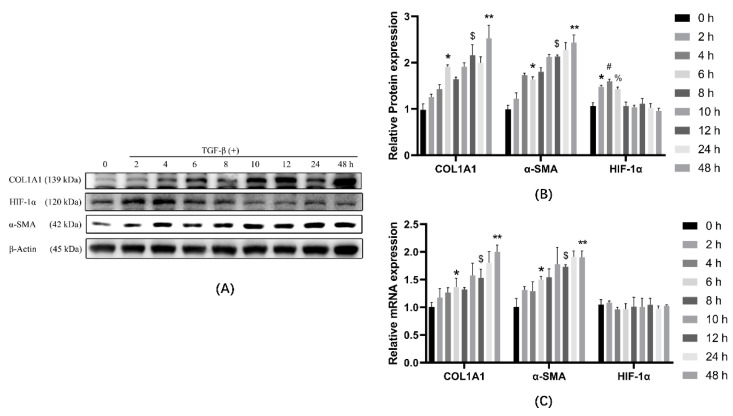
The effect of TGF-β1 on cell transdifferentiation was analyzed by comparing the expression of COL1A1, HIF-1α, and α-SMA using TGF-β1-treated cells for different times. (**A**) Western blot analysis of COL1A1, HIF-1α, and α-SMA in cells of different groups. β-actin was used as a loading control. (**B**) Quantitative analysis of COL1A1, HIF-1α, and α-SMA is shown in Figure 2A. (**C**) Cells were treated with TGF-β1 at different times, and mRNA was extracted from the treated groups. mRNA expression was determined by qPCR and normalized to β-actin. * *p* < 0.05 vs. 0 h group; ^#^ *p* < 0.05 vs. 2 h group; ^%^ *p* < 0.05 vs. 4 h group; ^$^ *p* < 0.05 vs. 6 h group; ** < 0.05 vs. 12 h group. ANOVA was used to compare multiple groups and pairwise comparisons were compared by LSD-t test. A value of *p* < 0.05 was considered to be statistically significant.

Based on the results of the above experiments, we concluded that COL1A1, α-SMA, and HIF-1α were significantly elevated by treatment with 7.5 ng/mL of TGF-β1. Therefore, we chose 7.5 ng/mL of TGF-β1 to treat the cells for 0 h, 2 h, 4 h, 6 h, 8 h, 10 h, 12 h, 24 h, and 48 h. Subsequently, the cells were collected for analysis. The results showed that HIF-1α protein expression increased after TGF-β1 treatment, reaching a maximum at 4 h (Figure 2A,B), and then gradually decreased, reaching baseline at 8 h (0 h) and then remaining stable. COL1A1 and α-SMA increased and accumulated during treatment. The mRNA results showed that COL1A1 and α-SMA increased continuously with increasing treatment time, and HIF-1α mRNA did not change. (Figure 2C).

### 3.3. HIF-1α and PSMAD3 Reached Maximum Expression at the Same Time

**Figure 3:**
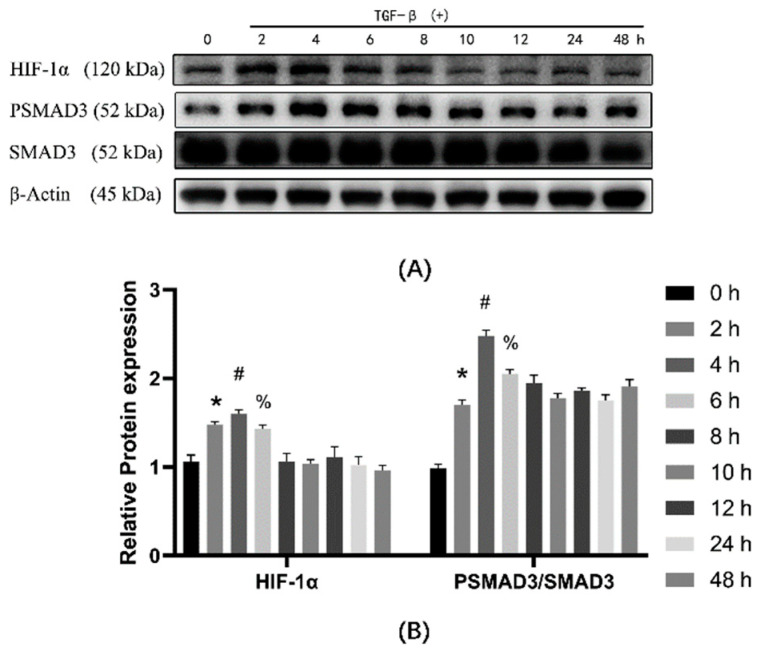
The effect of TGF-β1 on cell transdifferentiation was analyzed by comparing the expression of HIF-1α, SMAD3, and PSMAD3 using TGF-β1-treated cells. (**A**) Western blot analysis of HIF-1α, SMAD3, and PSMAD3 in cells of different groups. β-actin was used as a loading control. (**B**) Quantitative analysis of HIF-1α, PSMAD3/SMAD3 is shown in Figure 3A. * *p* < 0.05 vs. 0 h group; ^#^ *p* < 0.05 vs. 2 h group; ^%^ *p* < 0.05 vs. 4 h group. ANOVA was used to compare multiple groups and pairwise comparisons were compared by LSD-t test. A value of *p* < 0.05 was considered to be statistically significant.

The results of TGF-β1 treatment for different times (Figure 2B) show the result of the treatment with TGF-β1 for 2 h. The expression of HIF-1α, COL1A1, and α-SMA increased. It was reported that TGF-β1 promoted the phosphorylation of the SMAD3 gene and promoted the increase of α-SMA and COL1A1 expression. To investigate whether the alteration of HIF-1α is related to PSMAD3, we examined HIF-1α, PSMAD3, and SMAD3 and calculated the PSMAD3/SMAD3. The results (Figure 3B) show that after treatment with TGF-β1, SMAD3 expression decreased and PSMAD3 first increased and then decreased, reaching a maximum at 4 h. PSMAD3 is formed by SMAD3 phosphorylation, so it is affected by SMAD3 expression, and to eliminate the decrease in PSMAD3 caused by the decrease in SMAD3 expression after TGF-β1 treatment, we used PSMAD3/SMAD3. the results show that both HIF-1α expression and PSMAD3/SMAD3 increased and then decreased, with the highest expression at 4 h of TGF-β1 treatment.

### 3.4. HIF-1α Affects the Phosphorylation of SMAD3 and the Expression of COL1A1

**Figure 4:**
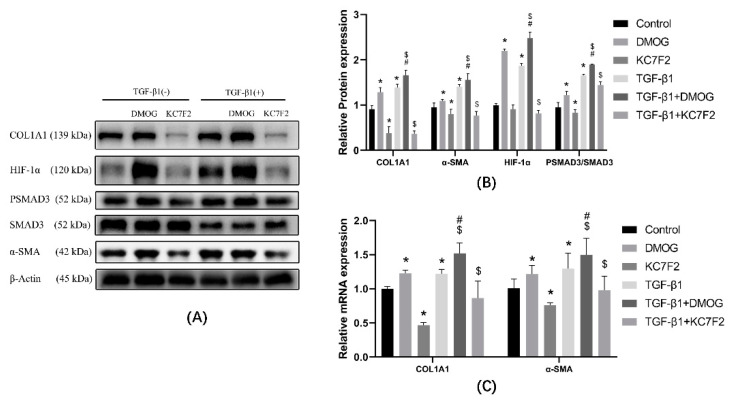
Cells were treated with DMSO, DMOG, and KC7F2 in the presence or absence of TGF-β1. mRNA and protein expression were examined to infer the degree of transdifferentiation. TGF-β1 was not present in the control group, the DMOG group, and the KC7F2 group. TGF-β1 was present in all of the TGF-β1 groups, the TGF-β1 + DMOG group, and the TGF-β1 + KC7F2 group. (**A**) Western blot analysis of COL1A1, HIF-1α, α-SMA, SMAD3, and PSMAD3 in cells of different groups. β-actin was used as loading control. (**B**) Quantitative analysis of COL1A1, HIF-1α, PSMAD3/SMAD3, and α-SMA is shown in Figure 4A. (**C**) Different reagents were used to treat different groups of cells, and mRNA was extracted from groups. mRNA expression was determined by qPCR and normalized to β-actin. * *p* < 0.05 vs. control group; ^#^ *p* < 0.05 vs. DMOG group; ^$^ *p* < 0.05 vs. TGF-β1 group. Results are expressed as means ± SD. ANOVA was used to compare multiple groups and pairwise comparisons were compared by LSD-t test. A value of *p* < 0.05 was considered to be statistically significant.

DMOG or KC7F2 was used to elevate or inhibit HIF-1α expression. Western blot or PCR was used to detect COL1A1, α-SMA, PSMAD3, and SMAD3 The protein results are shown in Figure 4A,B. Compared with the control group, the expression of α-SMA, COL1A1, and PSMAD3/SMAD3 were elevated in the DMOG group; the expression of COL1A1, α-SMA, and PSMAD3/SMAD3 were decreased in the KC7F2 group. Compared with the control group, the protein expression of COL1A1, α-SMA, and PSMAD3/SMAD3 were increased in the TGF-β1 group. Compared with the TGF-β1 group, COL1A1, α-SMA, and PSMAD3/SMAD3 expression were elevated in the TGF-β1 + DMOG group; COL1A1, α-SMA, PSMAD3/SMAD3 expression was decreased in the TGF-β1 + KC7F2 group. mRNA results showed (Figure 4C) that, compared with the control group, COL1A1 and α-SMA expression was elevated in the DMOG group; COL1A1 and α-SMA expression was decreased in the KF7F2 group; and COL1A1 and α-SMA expression was elevated in the TGF-β1 group. Compared with the TGF-β1 group, COL1A1 and α-SMA expression were increased in the TGF-β1 + DMOG group, and COL1A1 and α-SMA expression were decreased in the TGF-β1 + KC7F2 group.

### 3.5. Expression of α-SMA and Col1a Was Affected by Knockdown of HIF-1α Using siRNA

**Figure 5:**
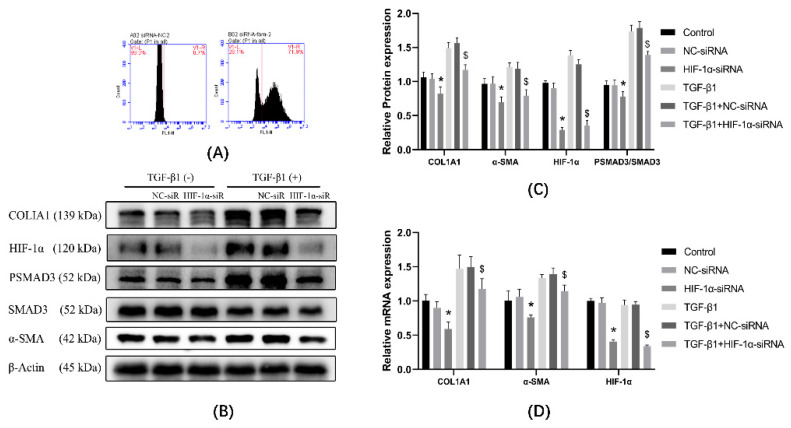
Cells were treated with NC-siRNA and HIF-1α-siRNA in the presence or absence of TGF-β1 and changes in mRNA and protein were examined to infer the degree of transdifferentiation. (**A**) Using flow cytometry, cell fluorescence intensity (transfection of siRNA that does not carry fluorescent groups) was measured, and 99.3% of the fluorescence intensity range was set as the threshold value (below which the cells were considered not to carry fluorescent groups), and transfection efficiency was calculated based on this threshold value. According to the threshold, cells exceeding the threshold were identified as successfully transfected with HIF-1α-siRNA (carrying fluorescent moieties). (HIF-1α-siRNA transfection efficiency was 71.9%) (**B**) Western blot analysis of COL1A1, HIF-1α,α-SMA, SMAD3, and PSMAD3 in cells of different groups. β-actin was used as loading control. (**C**) Quantitative analysis of α-SMA, COL1A1, PSMAD3/SMAD3, and HIF-1αis shown in Figure 5B. (**D**) mRNA was extracted from the different groups, determined by qPCR, and normalized to β-actin. * *p* < 0.05 vs. NC-siRNA group; ^$^ *p* < 0.05 vs. TGF-β1 + NC-siRNA group. Results are expressed as means ± SD. ANOVA was used to compare multiple groups and pairwise comparisons were compared by LSD-t test. A value of *p* < 0.05 was considered to be statistically significant.

Cells were transfected with HIF-1α-siRNA or NC-siRNA targeting HIF-1α-specific sequences (Figure 5A, transfection efficiency 71.9%). The protein expression results (Figure 5B,C) showed that, compared with the control group, the expression of α-SMA and COL1A1 were decreased in the HIF-1α-siRNA group, and the protein expression of α-SMA and COL1A1 were unchanged in the NC-siRNA group. Compared with the TGF-β1 group, the protein expression of α-SMA and COL1A1 were decreased in the TGF-β1 + HIF-1α-siRNA group, and the protein expression of α-SMA and COL1A1 were unchanged in the TGF-β1 + NC-siRNA group. mRNA results (Figure 5D) showed that, compared with the control group, the expression of α-SMA and COL1A1 were not changed in NC-siRNA group, and α-SMA and COL1A1 were decreased in the HIF-1α-siRNA group. Compared with the TGF-β1 group, the expression of α-SMA and COL1A1 were not changed in TGF-β1 + NC-siRNA group, and the expression of α-SMA and COL1A1 were decreased in the TGF-β1 + HIF-1α-siRNA group.

### 3.6. SIS3 Inhibits DMOG Induced Elevation of COL1A1

**Figure 6:**
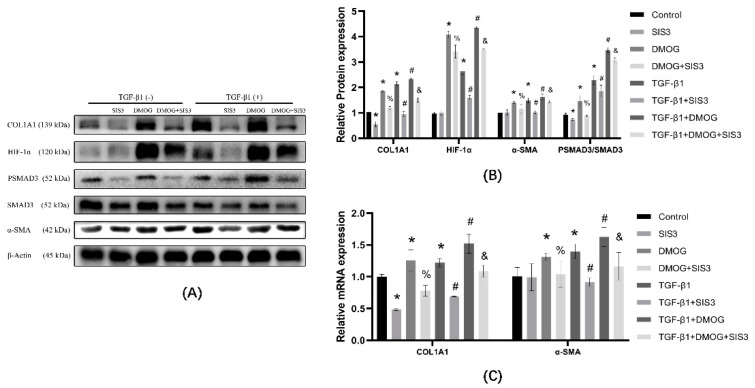
Cells were treated with DMSO, DMOG, SIS3, and SIS3 + DMOG in the presence or absence of TGF-β1 and changes in mRNA and protein were examined to infer the degree of transdifferentiation. (**A**) Western blot analysis of different groups COL1A1, HIF-1α, SMAD3, and PSMAD3. β-actin was used as loading control. (**B**) Quantitative analysis of COL1A1, HIF-1α, PSMAD3/SMAD3, and α-SMA is shown in Figure 6A. (**C**) Different reagents were used to treat different groups of cells, and mRNA was extracted. mRNA expression was determined by qPCR and normalized to β-actin. * *p* < 0.05 vs. control group; ^#^ *p* < 0.05 vs. TGF-β1; ^%^ *p* < 0.05 vs. DMOG; ^&^ *p* < 0.05 vs. TGF-β1 + DMOG group Results are expressed as means ± SD. ANOVA was used to compare multiple groups and pairwise comparisons were compared by LSD-t test. A value of *p* < 0.05 was considered to be statistically significant.

In the protein expression results shown (Figure 6A,B), compared with the control group, the expression of COL1A1 and PSMAD3/SMAD3 decreased in the SIS3 group, and the expression of α-SMA did not change significantly; COL1A1, α-SMA, HIF-1α, and PSMAD3/SMAD3 expression were elevated in the DMOG group. Compared with the DMOG group, the expression of COL1A1, α-SMA, and PSMAD3/SMAD3 were decreased in the DMOG + SIS3 group. Compared with TGF-β1 group, COL1A1, α-SMA, and PSMAD3/SMAD3 expression were decreased in TGF-β1 + SIS3 group; COL1A1, α-SMA, HIF-1α, PSMAD3/SMAD3 expression were increased in TGF-β1 + DMOG group. Compared with the TGF-β1 + DMOG group, the expression of COL1A1, α-SMA, and PSMAD3/SMAD3 were decreased in the TGF-β1 + DMOG + SIS3 group. The mRNA expression results show that (Figure 6C), compared with the control group, COL1A1, and α-SMA expression decreased in the SIS3 group, and COL1A1 and α-SMA expression increased in the DMOG group. Compared with the DMOG group, the expression of COL1A1 and α-SMA in the DMOG + SIS3 group were decreased. Compared with the TGF-β1 group, COL1A1 and α-SMA expression decreased in the TGF-β1 + SIS3 group, and COL1A1 and α-SMA expression increased in the TGF-β1 + DMOG group. Compared with the TGF-β1 + DMOG group, COL1A1 and α-SMA expression decreased in the TGF-β1 + DMOG + SIS3 group.

### 3.7. KC7F2 Alleviates Silica Particles Induced PF in Mice

**Figure 7:**
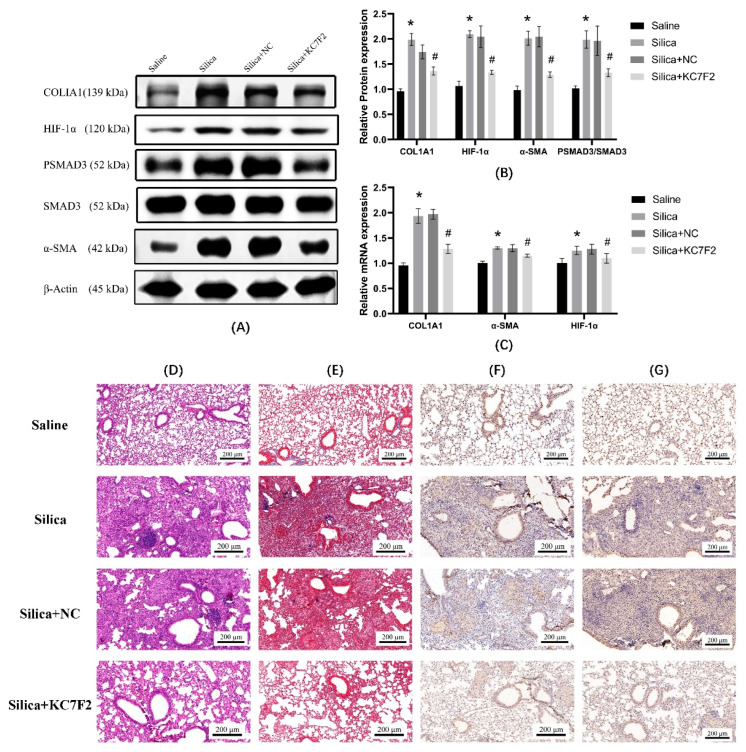
A mouse PF model was established by nonexposure tracheal drip in C57BL/6N mice using saline or silica. The extent of PF in mice was inferred by Western blot, PCR, and IHC. Saline group (saline tracheal drip), silica group (silica particles tracheal drip), silica + NC group (silica particles tracheal drip, using saline intraperitoneal injection), and silica + KC7F2 group (silica particles, using KFC7F intraperitoneal injection). (**A**) Western blot analysis of COL1A1, HIF-1α,α-SMA, SMAD3, and PSMAD3 in lung tissue of different groups. β-actin was used as loading control. (**B**) Quantitative analysis of COL1A1, HIF-1α, α-SMA PSMAD3/SMAD3 is shown in Figure 7A. (**C**) Quantitative analysis of the expression of mRNA. β-actin was used as an internal control. (**D**) H & E trichrome staining of mice lung tissue (**E**) Masson trichrome staining of mice lung tissue (**F**) IHC of COL1A1 protein. (**G**) IHC of HIF-1α protein. * *p* < 0.01 vs. saline group; ^#^ *p* < 0.01 vs. silica group. Results are expressed as means ± SD. ANOVA was used to compare multiple groups and pairwise comparisons were compared by LSD-t test. A value of *p* < 0.05 was considered to be statistically significant.

For 6-week-old C57BL/6N silica, silica + KC7F2, and silica + NC groups of mice were treated with 50 μL SiO_2_ suspension (50 mg/mL) by nonexposed tracheal drip to establish a silica-induced mouse PF model. Briefly, 50 μL saline was dripped by nonexposed tracheal drip for the saline group mice. There was no intervention for the saline and silica groups after nonexposed tracheal drip. KC7F2 group mice were injected intraperitoneally with KC7F2 solution (0.75 mg/Kg) at a frequency of once every 3 days. NC group mice were injected intraperitoneally with an equal amount of saline. The protein expression results show (Figure 7A,B) that, compared with the saline group, the expression of COL1A1, HIF-1α, α-SMA, and PSMAD3/SMAD3 were elevated in the tissues of mice in the silica and silica + NC groups. Compared with the silica group, the expression of COL1A1, HIF-1α, α-SMA, and PSMAD3/SMAD3 were decreased in the lung tissues of mice in the silica + KC7F2 group. The mRNA expression results show (Figure 7C) that, compared with the saline group, COL1A1, α-SMA, and HIF-1α expression were elevated in the silica group compared with the saline + NC group. COL1A1, α-SMA and HIF-1α mRNA expression were decreased in the silica + KC7F2 group compared with the silica group. The HE and Masson staining results (Figure 7D,E) show that, compared to the saline group, the alveolar structure was disrupted in the NC and silica groups, and the appearance of cellular nodules, collagen secretion increased, and fibroblasts increased. Compared with the silica group, the KC7F2 group showed a decrease in the number of cell nodules, a decrease in the degree of inflammatory infiltration, a decrease in collagen secretion, and a decrease in fibrosis in the lungs. IHC results (Figure 7F,G) show that, compared with the saline group, the expression levels of COL1A1 and HIF-1α were increased in the lung tissues of the silica and NC groups and clustered around the disrupted alveolar structures. Compared with the silica group, the expression of COL1A1 and HIF-1α in the KC7F2 group were reduced.

## 4. Discussion

This experiment showed that HIF-1α was associated with fibroblast transdifferentiation. HIF-1α affected fibroblast transdifferentiation through PSMAD3. Inhibition of HIF-1α reduced the elevation of COL1A1, PSMAD3, and α-SMA induced by TGF-β1. Subsequently, we validated this result in mice. HIF-1α protein was elevated in PF induced by silica in mice. The inhibition of HIF-1α by KC7F2 reduced the degree of fibrosis and decreased the expression of COL1A1 and α-SMA. In conclusion, silica-induced PF in mice was attenuated by KC7F2 through the inhibition of HIF-1α expression.

With the development of PF, the function of pulmonary gas exchange in patients with PF is seriously impaired. Therefore, reducing the secretion of EMC and enhancing the function of alveolar gas exchange, based on delaying the progression of fibrosis, is essential to treat the disease and relieve patient symptoms of dyspnea. Fibroblast transdifferentiation to form myofibroblasts and secretion of large amounts of ECM is an important process in silica-induced PF. Fibroblast transdifferentiation to form myofibroblasts and secretion of large amounts of ECM is an important process, and endogenous TGF-β1 plays an important role in silica-induced PF. TGF-β1 has been reported to play an important role as a transforming growth factor in liver fibrosis, kidney fibrosis, and PF. Some natural TGF-β1 inhibitors have been proven to be effective in inhibiting fibrosis. Natural sulfur-containing compounds, such as ovothiol A and taurine, can modulate redox-sensitive pathways such as the transforming growth factor-β pathway to slow down the fibrosis process [21]. These sulfur-containing natural compounds can be isolated from the diet or marine organisms, and combining diet with natural compounds with anti-fibrotic properties can be considered an effective therapeutic strategy to reverse fibrosis. Some researchers [22] studied the effect of ovothiol A isolated from sea urchin eggs on a mouse liver fibrosis model. The study induced liver fibrosis in mice by intraperitoneal injection of carbon tetrachloride (CCl4) and treated it with ovothiol A. The results showed that ovothiol A plays an anti-fibrosis role by regulating the expression and activity of membrane-bound γ-glutamyl-transpeptidase (GGT) and decreasing the expression of fibrosis markers such as TGF-β1, α-SMA, and tissue metalloproteinase inhibitor (TIMP-1). In this study, the results showed that, compared with the control group, HIF-1α, COL1A1, PSMAD3, and α-SMA expression were elevated in the TGF-β1 group. In silica-induced PF, HIF-1α expression was similarly elevated.

HIF-1α has been reported to be involved in metabolic, neoplastic, and fibrotic diseases such as myocardial fibrosis, hepatic fibrosis, and renal fibrosis [23,24,25,26,27] HIF-1α has been reported to be involved in the activation of the NOTCH signaling pathway in diseases such as Ang II-induced myocardial fibrosis and odontogenic keratotic cysts [28,29]. The expression of HIF-1α, α-SMA, and COL1 are elevated in cardiomyocytes overexpressing NOTCH3. In nonalcoholic fatty liver disease, HIF-1α has also been reported to promote liver fibrosis by acting on the PTEN/p65 pathway [30]. HIF-1α has also been reported to affect the EMT process by regulating the expression levels of Snail and β-catenin. In paraquat poisoning-induced PF, silencing of HIF-1α was effective in reducing the extent of PF [27]. In addition, HIF-1α is also considered to be a protein essential for activating tumor growth. It was reported that, in tumor-associated fibroblasts (CAFs), HIF-1α activates the NF-κB signaling pathway causing an increase in the expression level of CCL5, which promotes tumor growth [31]. Probucol is a cholesterol-lowering drug with potent antioxidant properties. It was reported that in bleomycin-induced PF, probucol inhibited EMT through the HIF-1α/TGF-β1 signaling pathway and attenuated the extent of PF. Our results showed that, compared with the silica group, the silica + KC7F2 group showed reduced expression of HIF-1α, COL1A1, α-SMA, and PSMAD3 and reduced lung fibrosis. Therefore, this study concluded that, for silica-induced PF, inhibition of HIF-1α could also play a role in reducing fibrosis. Although it has been reported that inhibition of HIF-1α in hepatic fibrotic cells reduces COL1, α-SMA expression, and the degree of fibrosis, it has not been indicated whether there is an association between HIF-1α and PSMAD3. PSMAD3 has been reported to be associated with fibrosis, and inhibition of PSMAD3 alleviates renal fibrosis [32]. TGF-β1 upregulates COL1A1 and α-SMA expression through PSMAD3 [10]. Our results showed that compared to the control group, PSMAD3, α-SMA, and COL1A1 were elevated in the DMOG group and TGF-β1 group. Compared with the TGF-β1 group, PSMAD3, α-SMA, and COL1A1 were decreased in the TGF-β1 + SIS3 group. Compared with the DMOG group, PSMAD3, α-SMA, and COL1A1 were reduced in the DMOG + SIS3 group. Therefore, this study concluded that HIF-1α regulates COL1A1 and α-SMA expression through PSMAD3.

Due to the presence of HIF-1α ODD, HIF-1α expression is affected by both production and degradation. The hydroxylation of HIF-1α ODD by PHD promoted the binding of pVHL to HIF-1α, allowing the proteasome to degrade HIF-1α. Therefore, HIF-1α levels can be regulated by modulating PHD activity or pVHL activity [33]. Prolyl hydroxylation is a key step in HIF-1α degradation. Hypoxia and inhibition of PHD activity by CoCl2 lead to elevated HIF-1α expression. Neotuberostemonine (NTS) treatment reversed the decrease in PHD activity induced by hypoxia or CoCl_2_ and reduced HIF-1α expression. NTS treatment reduced bleomycin-induced PF and ECM expression in mice [34]. Affecting the transcriptional or translational process of HIF-1α regulates the expression of HIF-1α and has been reported to be effective. For example, paeoniflorin inhibits HIF-1α translation by mTOR and attenuates CCL4-induced liver fibrosis in rats [35]. Rapamycin, an mTOR inhibitor, has been reported to inhibit TGF-β1-induced elevated COL1 expression through the mTOR/HIF-1α pathway, and this effect was reversed when HIF-1α expression was elevated [12]. Additionally, 2-Methoxyestradiol(2-ME)reduces HIF-1α protein levels through a translational-dependent pathway rather than affecting HIF-1α protein stability [36,37], and 2-ME reduced the increase in the expression of α-SMA, COL1, and COL3 caused by the increase in the NOTCH3 gene [38]. siRNA-specific silencing of NOTCH3 decreased the expression levels of α-SMA, COL1, and COL3. The expression of α-SMA, COL1, and COL3 increased when HIF-1α expression was elevated by treatment with DMOG. In this study, HIF-1α was inhibited by siRNA and KC7F2. KC7F2, an HIF-1α translation inhibitor, did not affect HIF-1α mRNA or the degradation process [39]. The results show that, compared with the TGF-β1 group, the expression of HIF-1α, α-SMA, and COL1A1 were decreased in TGF-β1 + HIF-1α-siRNA and TGF-β1 + KC7F2 groups. This suggests that reducing HIF-1α expression in different ways inhibited the expression of COL1A1 and α-SMA induced by TGF-β1. Therefore, we consider that different approaches to intervening in HIF-1a have similar effects.

The proliferation and transdifferentiation of fibroblasts, the secretion of ECM, and the repair of damaged tissues during PF give rise to energy requirements. When the oxygen-dependent tricarboxylic acid cycle does not supply enough energy to satisfy the energy demand, the body has to increase nonoxygen-dependent glycolysis to increase energy production [40,41]. Alveolar damage and ECM accumulation-induced hypoxia further enhance glycolysis. It was reported that the enzyme required for glycolysis is regulated by HIF-1α [42]. The generation of new blood vessels is very important in the repair process of damaged tissues. Vascular endothelial growth factor, a target gene of HIF-α [13], was considered the most potent endothelial-specific mitogen that plays an important role in the process of vascular remodeling. HIF-1α participates in multiple biological processes at different stages of PF; therefore, the role of HIF-1α in PF should be paid more attention. Targeting HIF-1α to inhibit PF may be a new approach to therapy for PF.

## 5. Conclusions

This study concluded that HIF-1α inhibition attenuated the TGF-β1-induced elevation of COL1A1 and α-SMA. In silica-induced PF mice, HIF-1α expression was elevated. Inhibition of HIF-1α using KC7F2 reduced COL1A1 and α-SMA expression and attenuated fibrosis in mouse lung tissue.

## Figures and Tables

**Table 1:** Sequences of the primers.

Gene	Forward, 5′-3′	Reverse, 5′-3′
COL1A1	TAAGGGTCCCCAATGGTGAGA	GGGTCCCTCGACTCCTACAT
α-SMA	GGCACCACTGAACCCTAAGG	ACAATACCAGTTGTACGTCCAGA
SMAD3	AGACGCCAGTTCTACCTCCAGTG	GCCAGCAGGGAAGTTAGTGTTCTC
HIF-1α	CTGCCACTGCCACCACAACTG	TGCCACTGTATGCTGATGCCTTAG
β-Actin	GTGCTATGTTGCTCTAGACTTCG	ATGCCACAGGATTCCATACC

## Data Availability

All data generated or analyzed during this study are included in this published article.
